# Supplementary material for: SPT6 promotes epidermal differentiation and blockade of an intestinal-like phenotype through control of transcriptional elongation
Source: Nat Commun. 2021 Feb 4;12:784. doi: 10.1038/s41467-021-21067-w (PMC7862286; doi:10.1038/s41467-021-21067-w)
Supplement: Supplementary file 1 — Supplementary Information [file 41467_2021_21067_MOESM1_ESM.pdf]

Supplementary Figure 1

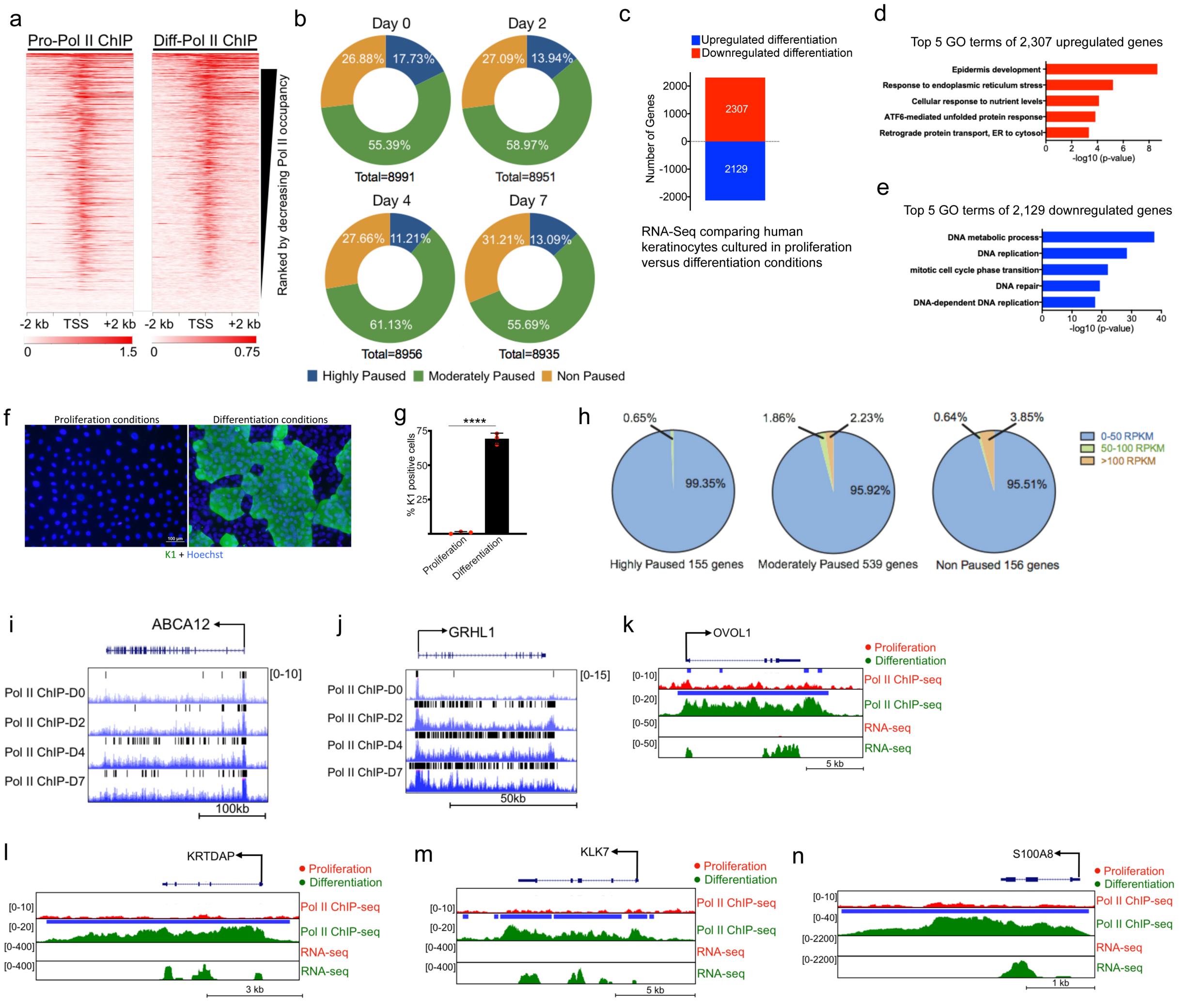

Supplementary Figure 1. A subset of induced epidermal differentiation genes are paused in proliferative cells and released upon differentiation.
(a) Heatmap of the RNA Pol II ChIP-Seq performed in proliferation (Pro) and differentiation (Diff) conditions. Pol II binding is shown ranked by decreasing Pol II occupancy. X-axis shows genic regions -2kb upstream to +2kb downstream of the transcription start site (TSS). N=3 independent experiments. **(b)** Donut chart of percentage of genes that are highly paused, moderately paused, non-paused during time-course of epidermal differentiation (days 0, 2, 4, and 7). Genes that contained significant Pol II binding (Pol II ChIP-Seq) during the timecourse were subjected to travel ratio analysis. **(c)** Bar graph summarizing the differentially expressed genes between proliferation and differentiation conditions. 2,307 genes were significantly upregulated and 2,129 genes were decreased during epidermal differentiation. **(d)** Gene ontology (GO) terms for the 2,307 genes upregulated during differentiation using hypergeometric distribution. **(e)** GO terms for the 2,129 genes downregulated during epidermal differentiation using hypergeometric distribution. **(f)** Immunofluorescent staining of early differentiation marker K1 (green) and Hoechst (blue) staining of nuclei. Primary human keratinocytes were cultured in proliferation conditions or differentiated for 3 days. n=3

independent experiments, scale bar = 100μm. **(g)** Quantitation of the percent of K1 positive cells from images taken in (f). Mean values are shown with error bars=SD. **** p <0.0001 (two-sided t-test). N=3 independent experiments. At least 100 cells were quantitated per experiment. **(h)** Breakdown of the percentage of highly paused (155 genes), moderately paused (539 genes), and non-paused (156 genes) genes that have RPKM values between 0-50 (blue), 50-100 (green), and greater than 100 (orange). **(i-j)** Gene tracks of two highly paused differentiation genes *ABCA12* (i) and *GRHL1* (j). Pol II ChIP-Seq tracks is shown during the timecourse of epidermal differentiation (D= day 0, day 2, day 4, and day 7 of differentiation). Y-axis shows reads per million and X-axis shows regions along the gene. Black bars over gene tracks show significant binding. **(k)** Gene track of a moderately paused differentiation gene *OVOL1.* Pol II ChIP-Seq tracks and RNA-Seq tracks are shown in proliferation (red) and differentiation (green) conditions. Y-axis shows reads per million and X-axis shows regions along the gene. Blue bars over gene track shows significant binding. **(l-n)** Gene tracks showing differentiation genes that have no RNA Pol II binding in proliferation conditions, but gain it upon differentiation. Pol II ChIP-Seq tracks and RNA-Seq tracks are shown in proliferation (red) and differentiation (green) conditions. Y-axis shows reads per million and X-axis shows regions along the gene. Blue bars over gene tracks show significant binding.

Supplementary Figure 2

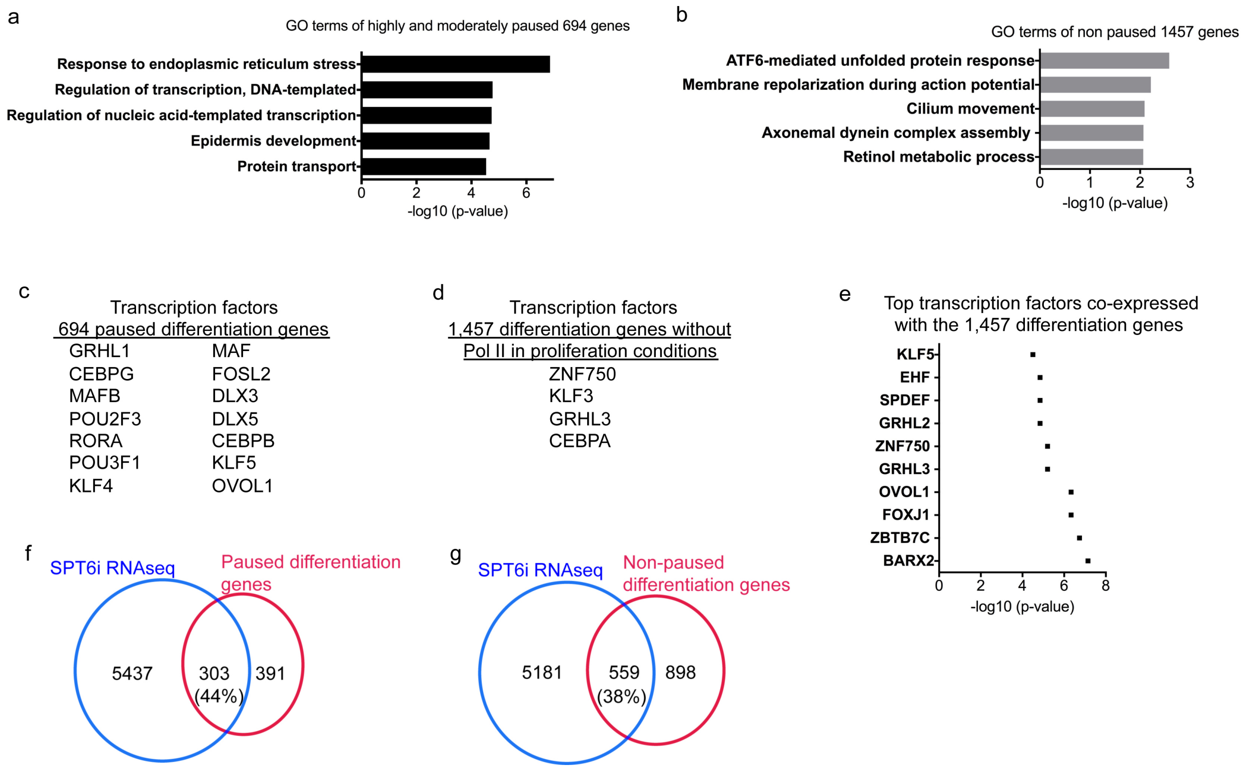

Supplementary Figure 2. Paused differentiation genes are enriched for transcription factors that may activate the non-paused differentiation genes.
(a) Gene Ontology (GO) terms for the 694 highly and moderately paused differentiation genes using hypergeometric distribution. **(b)** GO terms for the 1457 non-paused differentiation genes using hypergeometric distribution. **(c)** Known epidermal differentiation promoting transcription factors found in the 694 highly and moderately paused differentiation gene list. **(d)** Known epidermal differentiation promoting transcription factors found in the 1457 non-paused differentiation gene list. **(e)** The top 10 transcription factors co-expressed with the 1,457 non-paused differentiation genes using Enrichr. **(f)** Overlap of the SPT6 knockdown gene expression signature with the 694 highly and moderately paused differentiation genes. **(g)** Overlap of the SPT6 knockdown gene expression signature with the 1457 non-paused differentiation genes.

Supplementary Figure 3

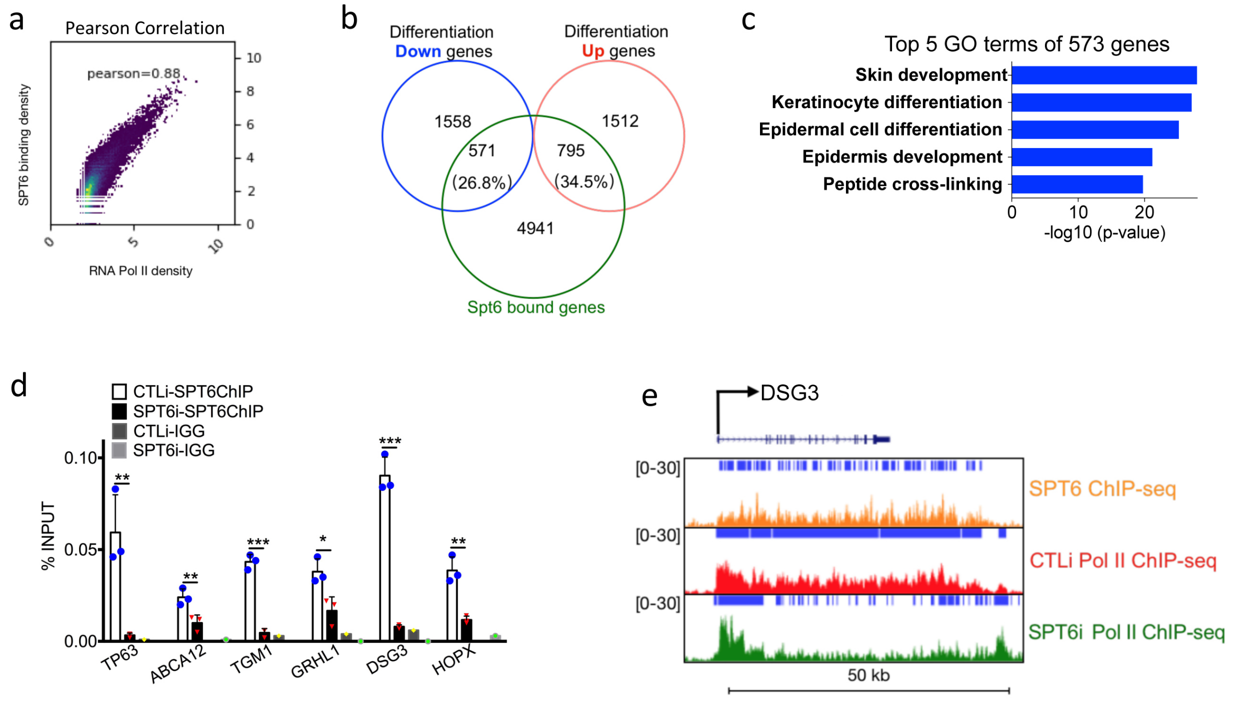

Supplementary Figure 3. SPT6 binding correlates with RNA Pol II and is necessary to promote transcription elongation.

(a) Pearson correlation between the SPT6 and RNA Pol II ChIP-Seq performed in differentiation conditions. **(b)** Venn diagram of SPT6 bound genes (SPT6 ChIP-Seq) with genes increased or decreased during epidermal differentiation. **(c)** Top 5 Gene Ontology (GO) terms for the 573 genes with reduced expression after SPT6 knockdown. These 573 genes are also bound directly by SPT6. **(d)** ChIP-qPCR of SPT6 pulldown in CTLi and SPT6i keratinocytes (day 3 differentiation). Each pulldown was normalized to its respective input and enrichment shown as a percent of input. IGG pulldowns from CTLi and SPT6i cells were used as a negative control. N=3 independent experiments. Mean values are shown with error bars=SD. **=0.0092 for TP63, **= 0.0186 for ABCA12, ***= 0.0001 for TGM1, *=0.0231 for GRHL1, ***=0.0001 for DSG3, **=0.0036 for HOPX (two-sided t-test). **(e)** Gene track of differentiation gene *DSG3*. SPT6 ChIP-Seq in differentiation conditions is shown in orange. CTLi (red) and SPT6i (green) RNA Pol II ChIP-Seq in differentiation conditions are also shown. Y-axis shows reads per million and blue bar over gene tracks represent significant peaks.

Supplementary Figure 4

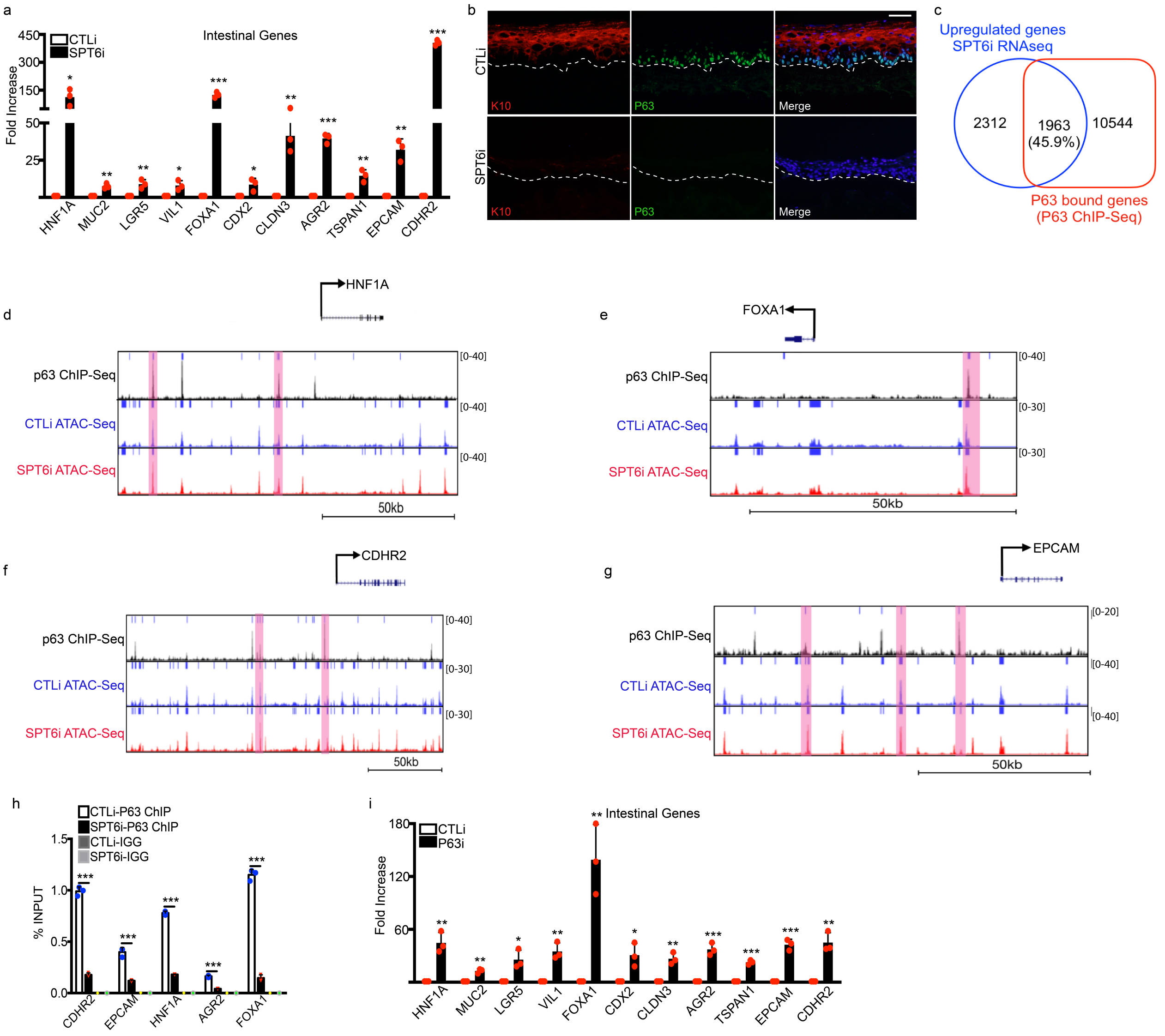

Supplementary Figure 4. SPT6 is necessary to suppress an intestinal fate through the epidermal master transcription factor, P63

(a) RT-qPCR quantifying the relative mRNA expression levels of intestinal genes in regenerated human epidermis (day 6) in CTLi and SPT6i samples. qPCR values were normalized to *L32*. n=3 independent experiments.*=0.0128 for HNF1A, **=0.0027 for MUC2, **= 0.0095 for LGR5, *= 0.0197 for VIL1, ***= 0.0001 for FOXA1, *= 0.0332 for CDX2, **=0.0039 for CLDN3, ***= 0.0001 for AGR2, **= 0.0045 for TSPAN1, **= 0.0017 for EPCAM, ***= 0.0001 for CDHR2. **(b)** Immunofluorescent staining of early differentiation marker K10 (red) and transcription factor P63 (green) in day 6 regenerated human epidermis treated with control (CTLi) or siRNAs targeting SPT6 (SPT6i). Merged image includes Hoechst staining of nuclei. n=3 independent experiments. Scale bar = 60μm. **(c)** Overlap of the 4,275 genes increased in expression upon SPT6 knockdown with the 12,507 genes that P63 binds (identified by P63 ChIP-Seq). **(d-g)** Gene tracks showing intestinal genes *HNF1A* (d), *FOXA1* (e), *CDHR2* (f) and *EPCAM* (g). ATAC-Seq signals for CTLi (blue) and SPT6i (red) are shown. P63 ChIP-Seq is shown in black. Regions marked in pink denote P63 bound regions with increased chromatin accessibility upon SPT6 depletion. Y-axis represents reads per million. X-axis denotes regions along the gene. **(h)** ChIP-

qPCR of P63 pulldown in CTLi and SPT6i keratinocytes (day 3 differentiation). Each pulldown was normalized to its respective input and enrichment shown as a percent of input. IGG pulldowns from CTLi and SPT6i cells were used as a negative control. n=3 independent experiments. **(i)** RT-qPCR quantifying the relative mRNA expression levels of intestinal genes in regenerated human epidermis (day 6) in CTLi and P63i samples. qPCR values were normalized to *L32*. n=3 independent experiments.**=0.0032 for HNF1A, **=0.0011 for MUC2, *=0.0167 for LGR5, **= 0.0036 for VIL1, **= 0.0040 for FOXA1, *= 0.0194 for CDX2, **=0.0026 for CLDN3, ***=0.0009 for AGR2, ***= 0.0001 for TSPAN1, ***=0.0003 for EPCAM, **= 0.0025 for CDHR2. All bar graphs show mean values with error bars=SD. *$\boldsymbol{p}$ <0.05, ** $\boldsymbol{p}$ <0.01, *** $\boldsymbol{p}$ <0.001 (two-sided t-test for 4a,h-i).
