# Supplementary material for: SPT6 promotes epidermal differentiation and blockade of an intestinal-like phenotype through control of transcriptional elongation
Source: Nat Commun. 2021 Feb 4;12:784. doi: 10.1038/s41467-021-21067-w (PMC7862286; doi:10.1038/s41467-021-21067-w)
Supplement: Supplementary file 3 — Reporting Summary [file 41467_2021_21067_MOESM3_ESM.pdf]

Corresponding author(s): George Sen

Last updated by author(s): Jan 3, 2021

Reporting Summary

Nature Research wishes to improve the reproducibility of the work that we publish. This form provides structure for consistency and transparency in reporting. For further information on Nature Research policies, see our Editorial Policies and the Editorial Policy Checklist.

Statistics

For all statistical analyses, confirm that the following items are present in the figure legend, table legend, main text, or Methods section.

n/a	Confirmed	
[ ]	[x]	The exact sample size (*n*) for each experimental group/condition, given as a discrete number and unit of measurement
[ ]	[x]	A statement on whether measurements were taken from distinct samples or whether the same sample was measured repeatedly
[ ]	[x]	The statistical test(s) used AND whether they are one- or two-sided<br>*Only common tests should be described solely by name; describe more complex techniques in the Methods section.*
[ ]	[x]	A description of all covariates tested
[ ]	[x]	A description of any assumptions or corrections, such as tests of normality and adjustment for multiple comparisons
[ ]	[x]	A full description of the statistical parameters including central tendency (e.g. means) or other basic estimates (e.g. regression coefficient) AND variation (e.g. standard deviation) or associated estimates of uncertainty (e.g. confidence intervals)
[ ]	[x]	For null hypothesis testing, the test statistic (e.g. *F*, *t*, *r*) with confidence intervals, effect sizes, degrees of freedom and *P* value noted<br>*Give P values as exact values whenever suitable.*
[x]	[ ]	For Bayesian analysis, information on the choice of priors and Markov chain Monte Carlo settings
[x]	[ ]	For hierarchical and complex designs, identification of the appropriate level for tests and full reporting of outcomes
[x]	[ ]	Estimates of effect sizes (e.g. Cohen's *d*, Pearson's *r*), indicating how they were calculated

Our web collection on statistics for biologists contains articles on many of the points above.

Software and code

Policy information about availability of computer code

Data collection	**Code availability statement:** The code for calculating travel ratio has been upload to github and can be found at the following address: https://github.com/maxabruzzi/travelRatio with **DOI of http://doi.org/10.5281/zenodo.4383281.**
Data analysis	Bar plots were drawn using GraphPad Prism v7. Software used for RNA-Seq and ChIP-Seq analysis include: TopHat2.1.1 (Kim, D., Pertea, G., Trapnell, C., Pimentel, H., Kelley, R., and Salzberg, S.L. (2013). TopHat2.1.1: accurate alignment of transcriptomes in the presence of insertions, deletions and gene fusions.Genome biology 14), MACS2 version 2.2.4 (Zhang, Y., Liu, T., Meyer, C.A., Eeckhoute, J., Johnson, D.S., Bernstein, B.E., Nusbaum, C., Myers, R.M., Brown, M., Li, W., et al. (2008). Model-based analysis of ChIP-Seq (MACS).Genome biology 9))., seqMINER (Ye, T., Krebs, A.R., Choukrallah, M.A., Keime, C., Plewniak, F., Davidson, I., and Tora, L. (2011). seqMINER: an integrated Ch IP-seq data interpretation platform. Nucleic Acids Res 39), Enrichr (Kuleshov, M.V., Jones, M.R., Rouillard, A.D., Fernandez, N.F., Duan, Q., Wang, Z., S.L., Koplev, S., Jagodnik .M., Lachmann, A., et al. (2016). Enrichr: a comprehensive gene set enrichment analysis web server 2016 update. Nucleic Acids Res 44), PartekGenomics Suite 6.0 (http://www.partek.com/partek-genomics-suite/), Kundaje ChIP-seq pipeline (https://github.com/kundajelab/chipseq_pipeline). Pearson correlation density plot was generated using deeptools(version 3.3.1) plot correlation (Ramirez F, Dundar F, Diehl S,Gruning BA, Manke T. deep Tools: a flexible platform for exploring deep-sequencing data. Nucleic Acids Res 2014;42(Web Server issue):W187- 91). De novo motif analysis was performed using HOMER version 4.10.4 (Heinz S, Benner C, Spann N, Bertolino E, Lin YC, Laslo P, et al. Simple combinations of lineage-determining transcription factors prime cis-regulatory elements required for macrophage and B cell identities. Mol Cell 2010;38(4):576-89.). Ngsplot version 2.61 was used to generate metagene plot(Shen L, Shao N, Liu X, Nestler E. ngs.plot: Quick mining and visualization of next-generation sequencing data by integrating genomic databases. BMC genomics 2014;15:284.).

For manuscripts utilizing custom algorithms or software that are central to the research but not yet described in published literature, software must be made available to editors and reviewers. We strongly encourage code deposition in a community repository (e.g. GitHub). See the Nature Research guidelines for submitting code & software for further information.

Data

Policy information about availability of data

All manuscripts must include a data availability statement. This statement should provide the following information, where applicable:

- Accession codes, unique identifiers, or web links for publicly available datasets
- A list of figures that have associated raw data
- A description of any restrictions on data availability

The datasets generated from this study including RNA-Seq, ChIP-Seq, and ATAC-Seq data has been deposited in GEO (GSE153129) at https://www.ncbi.nlm.nih.gov/geo/query/acc.cgi?acc=GSE153129.

Field-specific reporting

Please select the one below that is the best fit for your research. If you are not sure, read the appropriate sections before making your selection.

☒ Life sciences ☐ Behavioural & social sciences ☐ Ecological, evolutionary & environmental sciences

For a reference copy of the document with all sections, see nature.com/documents/nr-reporting-summary-flat.pdf

Life sciences study design

All studies must disclose on these points even when the disclosure is negative.

Sample size	No sample-size calculations were performed. Sample size was determined to be adequate based on the magnitude and consistency of measurable differences between groups.Sample sizes were not predetermined based on statistical methods, but were chosen according to the standards of the field (at least three independent biological replicates for each condition), which generated a sufficient number of single-molecule trajectories and gave sufficient statistics for the effect sizes of interest.
Data exclusions	Data were only excluded for failed experiments resulting from technical issues, such as uneven loading of gels such as Figure 2b.
Replication	Reported results were consistently replicated. Results were at least performed 3 independent times and showed similar results.
Randomization	No randomization is applied. No randomization was necessary for this study because investigators were comparing DNA or RNA samples under well controlled conditions. No human or animal subjects were used in the study.
Blinding	Investigators were not blinded to group allocation. Blinding is not necessary because the results are quantitative and did not require subjective judgment or interpretation.

Reporting for specific materials, systems and methods

We require information from authors about some types of materials, experimental systems and methods used in many studies. Here, indicate whether each material, system or method listed is relevant to your study. If you are not sure if a list item applies to your research, read the appropriate section before selecting a response.

Materials & experimental systems

n/a	Involved in the study	
☐	☒	Antibodies
x	☐	Eukaryotic cell lines
x	☐	Palaeontology and archaeology
x	☐	Animals and other organisms
x	☐	Human research participants
x	☐	Clinical data
x	☐	Dual use research of concern

Methods

n/a	Involved in the study	
☐	☒	ChIP-seq
X	☐	Flow cytometry
x	☐	MRI-based neuroimaging

Antibodies

Antibodies used	P63 (Abcam: Ab124762), FOXA1 at 1:1000, Loricrin at 1:1000 RNA Pol II antibody (Active Motif: 91151) ,Keratin 10 (Abcam: ab9025) at 1:500, Filaggrin (Abcam: ab3137) at 1:200, MKi67 (Abcam: ab16667) at 1:300, keratin 1 (Biolegend: 905201), Beta-Actin (Santa Cruz,sc47778) ELL(Cell Signaling Technology:14468), SPT5 (Bethyl: A300-869A), SPT6 (Bethyl: A300-801A), donkey anti-mouse (LI-COR:926-32212), donkey anti-rabbit (LI-COR:926-68073), Alexa 555 goat anti-mouse IgG (ThermoFisher A11029), Alexa 488 donkey anti-rabbit IgG (ThermoFisher A21206)
Validation	The P63 antibody has been thoroughly validated by the manufacturer for Western blot, Immunofluorescence, flowcytometry. This information can be found at https://www.abcam.com/p63-antibody-epr5701-ab124762.html.<br><br>The FOXA1 antibody has been thoroughly validated by the manufacturer for Western blot, Immunofluorescence, IP, ChIP. This information can be found at https://www.cellsignal.com/products/primary-antibodies/foxa1-hnf3a-e7e8w-rabbit-mab/53528

The RNA Pol II has been thoroughly validated by the manufacturer for Western blot, Immunofluorescence, ChIP-Seq. This information can be found at https://www.activemotif.com/catalog/details/91151/abflex-rna-pol-ii-antibody-rab.

The beta-actin antibody has been validated by the manufacturer for Western blotting and has also received 352 reviews. More information can be found at: https://www.scbt.com/scbt/product/beta-actin-antibody-c4.

The Keratin 1, Filaggrin, MKI67, and Keratin 10 antibodies are commonly used in immunofluorescence and has been cited in numerous publications as well as validated on the manufacturer's website:
Keratin 1: https://www.biolegend.com/en-ie/products/keratin-1-polyclonal-antibody-purified-10951
MKI67: https://www.abcam.com/ki67-antibody-sp6-ab16667.html
Keratin 10: https://www.abcam.com/cytokeratin-10-antibody-rkse60-ab9025.html
Loricrin: https://www.abcam.com/loricrin-antibody-epr71492-c-terminal-ab198994.html
The SPT6 antibody has been validated by the manufacturer for western blot, immunoprecipitation and immunohistochemistry and has been cited 7 times. More information can be found at https://www.bethyl.com/product/A300-801A/SUPT6H+Antibody.
The SPT5 antibody has been validated by the manufacturer for Western blot and immunoprecipitation and has been cited 4 times. More information can be be found at https://www.bethyl.com/product/A300-869A/SUPT5H+Antibody.
The ELL antibody has been validated by the manufacturer for western blot, immunoprecipitation, and ChIP. It has been cited 1 time. More information can be found at https://www.cellsignal.com/products/primary-antibodies/ell-d7n6u-rabbit-mab/14468?site-search-type=Products&N=4294956287&Ntt=ell&fromPage=plp.

Palaeontology and Archaeology

Specimen provenance | *Provide provenance information for specimens and describe permits that were obtained for the work (including the name of the issuing authority, the date of issue, and any identifying information).*

Specimen deposition | *Indicate where the specimens have been deposited to permit free access by other researchers.*

Dating methods | *If new dates are provided, describe how they were obtained (e.g. collection, storage, sample pretreatment and measurement), where they were obtained (i.e. lab name), the calibration program and the protocol for quality assurance OR state that no new dates are provided.*

☐ Tick this box to confirm that the raw and calibrated dates are available in the paper or in Supplementary Information.

Ethics oversight | *Identify the organization(s) that approved or provided guidance on the study protocol, OR state that no ethical approval or guidance was required and explain why not.*

Note that full information on the approval of the study protocol must also be provided in the manuscript.

Animals and other organisms

Policy information about studies involving animals; ARRIVE guidelines recommended for reporting animal research

Laboratory animals | *For laboratory animals, report species, strain, sex and age OR state that the study did not involve laboratory animals.*

Wild animals | *Provide details on animals observed in or captured in the field; report species, sex and age where possible. Describe how animals were caught and transported and what happened to captive animals after the study (if killed, explain why and describe method; if released, say where and when) OR state that the study did not involve wild animals.*

Field-collected samples | *For laboratory work with field-collected samples, describe all relevant parameters such as housing, maintenance, temperature, photoperiod and end-of-experiment protocol OR state that the study did not involve samples collected from the field.*

Ethics oversight | *Identify the organization(s) that approved or provided guidance on the study protocol, OR state that no ethical approval or guidance was required and explain why not.*

Note that full information on the approval of the study protocol must also be provided in the manuscript.

Human research participants

Policy information about studies involving human research participants

Population characteristics | *Describe the covariate-relevant population characteristics of the human research participants (e.g. age, gender, genotypic information, past and current diagnosis and treatment categories). If you filled out the behavioural & social sciences study design questions and have nothing to add here, write "See above."*

Recruitment | *Describe how participants were recruited. Outline any potential self-selection bias or other biases that may be present and how these are likely to impact results.*

Ethics oversight | *Identify the organization(s) that approved the study protocol.*

Note that full information on the approval of the study protocol must also be provided in the manuscript.

Clinical data

Policy information about clinical studies

All manuscripts should comply with the ICMJEguidelines for publication of clinical research and a completedCONSORT checklist must be included with all submissions.

Clinical trial registration	*Provide the trial registration number from ClinicalTrials.gov or an equivalent agency.*
Study protocol	*Note where the full trial protocol can be accessed OR if not available, explain why.*
Data collection	*Describe the settings and locales of data collection, noting the time periods of recruitment and data collection.*
Outcomes	*Describe how you pre-defined primary and secondary outcome measures and how you assessed these measures.*

Dual use research of concern

Policy information about dual use research of concern

Hazards

Could the accidental, deliberate or reckless misuse of agents or technologies generated in the work, or the application of information presented in the manuscript, pose a threat to:

No	Yes	
☒	☐	Public health
☒	☐	National security
☒	☐	Crops and/or livestock
☒	☐	Ecosystems
☒	☐	Any other significant area

Experiments of concern

Does the work involve any of these experiments of concern:

No	Yes	
☒	☐	Demonstrate how to render a vaccine ineffective
☒	☐	Confer resistance to therapeutically useful antibiotics or antiviral agents
☒	☐	Enhance the virulence of a pathogen or render a nonpathogen virulent
☒	☐	Increase transmissibility of a pathogen
☒	☐	Alter the host range of a pathogen
☒	☐	Enable evasion of diagnostic/detection modalities
☒	☐	Enable the weaponization of a biological agent or toxin
☒	☐	Any other potentially harmful combination of experiments and agents

ChIP-seq

Data deposition

☒ Confirm that both raw and final processed data have been deposited in a public database such as GEO.

☒ Confirm that you have deposited or provided access to graph files (e.g. BED files) for the called peaks.

Data access links<br>*May remain private before publication.*	All ChIP-seq files (raw data and BED files) have been deposited in the GEO database (accession GSE153129 as stated in the Methods section of the manuscript. The secure token to access these files is the following: whijeieqlduzjqb
Files in database submission	SPT6_CHIPseq_rep1<br>SPT6_CHIPseq_rep2

Input_for_SPT6_CHIPseq
Proliferation_Pol2_CHIPseq_rep1
Proliferation_Pol2_CHIPseq_rep2
Proliferation_Pol2_CHIPseq_rep3
Differentiation_Pol2_CHIPseq_rep1
Differentiation_Pol2_CHIPseq_rep2
Differentiation_Pol2_CHIPseq_rep3
SPT6i_Pol2_CHIPseq_rep1
SPT6i_Pol2_CHIPseq_rep2
Input_SPT6Pol2CHIP
Input_Pro_CHIP
Input_Diff_CHIP

Genome browser session (e.g. UCSC)

http://genome.ucsc.edu/cgi-bin/hgTracks?db=hg19&hubUrl=http://oncogx.ucsd.edu/projects/SPT6_new/hub19.txt

Methodology

Replicates

2 replicates for SPT6 ChIP, 3 replicates for RNA Pol2 ChIP in proliferating cells, 3 replicates for RNA Pol2 ChIP in differentiated cells, 2 replicates for Pol2 ChIPseq in SPT6i cells

Sequencing depth

Between 17 and 40 million reads were retained per library following quality filtering (details provided in Methods).

Antibodies

The following antibodies were used: SPT6 (Bethyl: A300-801A), RNA Pol •(Active motif: 91151) , Rabbit IgG (Millipore: 12-370) and mouse IgG (Abcam: ab18413).

Peak calling parameters

Upon alignment and deduplication, the peak-calling was then carried out by MACS2.2.4 with default setting (4-fold and P-value < 0.00001).

Data quality

All ChIP-seq dataset passed the NSC and RSC score standard according to ENCODE.

Software

The ChIP-seq reads were processed by the ENCODE Transcription Factor and Histone ChIP-Seq processing pipeline (https://github.com/ENCODE-DCC/chip-seq-pipeline2) on our local workstation. The reads were first trimmed based on quality score before alignment to reference hg19; Upon alignment and deduplication, the peak-calling was then carried out by MACS2.2.4 with a cutoff q-value of 0.05. The heatmaps for the ChIP-Seq data were generated using ngs.plot (version 2.61). Gene tracks were visualized using UCSC genome browser along with annotation tracks.

Flow Cytometry

Plots

Confirm that:

☐ The axis labels state the marker and fluorochrome used (e.g. CD4-FITC).

☐ The axis scales are clearly visible. Include numbers along axes only for bottom left plot of group (a 'group' is an analysis of identical markers).

☐ All plots are contour plots with outliers or pseudocolor plots.

☐ A numerical value for number of cells or percentage (with statistics) is provided.

Methodology

Sample preparation

Describe the sample preparation, detailing the biological source of the cells and any tissue processing steps used.

Instrument

Identify the instrument used for data collection, specifying make and model number.

Software

Describe the software used to collect and analyze the flow cytometry data. For custom code that has been deposited into a community repository, provide accession details.

Cell population abundance

Describe the abundance of the relevant cell populations within post-sort fractions, providing details on the purity of the samples and how it was determined.

Gating strategy

Describe the gating strategy used for all relevant experiments, specifying the preliminary FSC/SSC gates of the starting cell population, indicating where boundaries between "positive" and "negative" staining cell populations are defined.

☐ Tick this box to confirm that a figure exemplifying the gating strategy is provided in the Supplementary Information.

Magnetic resonance imaging

Experimental design

Design type

Indicate task or resting state; event-related or block design.

Design specifications | *Specify the number of blocks, trials or experimental units per session and/or subject, and specify the length of each trial or block (if trials are blocked) and interval between trials.*

Behavioral performance measures | *State number and/or type of variables recorded (e.g. correct button press, response time) and what statistics were used to establish that the subjects were performing the task as expected (e.g. mean, range, and/or standard deviation across subjects).*

Acquisition

Imaging type(s) | *Specify: functional, structural, diffusion, perfusion.*

Field strength | *Specify in Tesla*

Sequence & imaging parameters | *Specify the pulse sequence type (gradient echo, spin echo, etc.), imaging type (EPI, spiral, etc.), field of view, matrix size, slice thickness, orientation and TE/TR/flip angle.*

Area of acquisition | *State whether a whole brain scan was used OR define the area of acquisition, describing how the region was determined.*

Diffusion MRI ☐ Used ☐ Not used

Preprocessing

Preprocessing software | *Provide detail on software version and revision number and on specific parameters (model/functions, brain extraction, segmentation, smoothing kernel size, etc.).*

Normalization | *If data were normalized/standardized, describe the approach(es): specify linear or non-linear and define image types used for transformation OR indicate that data were not normalized and explain rationale for lack of normalization.*

Normalization template | *Describe the template used for normalization/transformation, specifying subject space or group standardized space (e.g. original Talairach, MNI305, ICBM152) OR indicate that the data were not normalized.*

Noise and artifact removal | *Describe your procedure(s) for artifact and structured noise removal, specifying motion parameters, tissue signals and physiological signals (heart rate, respiration).*

Volume censoring | *Define your software and/or method and criteria for volume censoring, and state the extent of such censoring.*

Statistical modeling & inference

Model type and settings | *Specify type (mass univariate, multivariate, RSA, predictive, etc.) and describe essential details of the model at the first and second levels (e.g. fixed, random or mixed effects; drift or auto-correlation).*

Effect(s) tested | *Define precise effect in terms of the task or stimulus conditions instead of psychological concepts and indicate whether ANOVA or factorial designs were used.*

Specify type of analysis: ☐ Whole brain ☐ ROI-based ☐ Both

Statistic type for inference (See Eklund et al. 2016) | *Specify voxel-wise or cluster-wise and report all relevant parameters for cluster-wise methods.*

Correction | *Describe the type of correction and how it is obtained for multiple comparisons (e.g. FWE, FDR, permutation or Monte Carlo).*

Models & analysis

n/a	Involved in the study
☐	☐ Functional and/or effective connectivity
☐	☐ Graph analysis
☐	☐ Multivariate modeling or predictive analysis

Functional and/or effective connectivity | *Report the measures of dependence used and the model details (e.g. Pearson correlation, partial correlation, mutual information).*

Graph analysis | *Report the dependent variable and connectivity measure, specifying weighted graph or binarized graph, subject- or group-level, and the global and/or node summaries used (e.g. clustering coefficient, efficiency, etc.).*

Multivariate modeling and predictive analysis | *Specify independent variables, features extraction and dimension reduction, model, training and evaluation metrics.*
