# Supplementary material for: SPT6 promotes epidermal differentiation and blockade of an intestinal-like phenotype through control of transcriptional elongation
Source: Nat Commun. 2021 Feb 4;12:784. doi: 10.1038/s41467-021-21067-w (PMC7862286; doi:10.1038/s41467-021-21067-w)
Supplement: Supplementary file 4 — Description of Additional Supplementary Files [file 41467_2021_21067_MOESM4_ESM.pdf]

Description of Additional Supplementary Files

File Name:Supplementary Data 1
Description: ChIP-Seq of RNA Polymerase II in proliferating primary human keratinocytes. Peak start and end coordinates are shown. Peaks were also mapped back to the nearest gene.

File Name: Supplementary Data 2
Description: ChIP-Seq of RNA Polymerase II in differentiated primary human keratinocytes. Cells were differentiated in full confluence in the presence of 1.2mM calcium for 3 days. Peak start and end coordinates are shown. Peaks were also mapped back to the nearest gene.

File Name: Supplementary Data 3
Description: RNA-Seq data comparing proliferating and differentiated primary human keratinocytes. Differentially expressed genes are shown along with fold change values.

File Name: Supplementary Data 4
Description:RNA-Seq data comparing Control (CTLi) and SPT6 knockdown (SPT6i) primary human keratinocytes. CTLi and SPT6i cells were differentiated in full confluence in the presence of 1.2mM calcium for 3 days. Differentially expressed genes are shown along with fold change values.

File Name: Supplementary Data 5
Description: ChIP-Seq of SPT6 in differentiated primary human keratinocytes. Cells were differentiated in full confluence in the presence of 1.2mM calcium for 3 days. Peak start and end coordinates are shown. Peaks were also mapped back to the nearest gene.

File Name: Supplementary Data 6
Description: ChIP-Seq of RNA Polymerase II in differentiated primary human keratinocytes. Cells were differentiated in full confluence in the presence of 1.2mM calcium for 3 days. Peak start and end coordinates are shown. Peaks were also mapped back to the nearest gene. Supplementary Data 6a shows control samples. Supplementary Data 6b shows SPT6 knockdown samples.

File Name: Supplementary Data 7
Description: ATAC-Seq of control and SPT6 knockdown cells.Cells were differentiated in full confluence in the presence of 1.2mM calcium for 3 days. Peak start and end coordinates are shown. Peaks were also mapped back to the nearest gene. Supplementary Data 7a shows regions that lost chromatin accessibility upon SPT6 knockdown. Supplementary Data 7b shows regions that gain chromatin accessibility upon SPT6 knockdown.

File Name: Supplementary Data 8
Description: This dataset lists all of the siRNA, RT-qPCR primers, and ChIP primers used in this study.
